# Novel virus-like nanoparticle vaccine effectively protects animal model from SARS-CoV-2 infection

**DOI:** 10.1371/journal.ppat.1009897

**Published:** 2021-09-07

**Authors:** Qibin Geng, Wanbo Tai, Victoria K. Baxter, Juan Shi, Yushun Wan, Xiujuan Zhang, Stephanie A. Montgomery, Sharon A. Taft-Benz, Elizabeth J. Anderson, Audrey C. Knight, Kenneth H. Dinnon, Sarah R. Leist, Ralph S. Baric, Jian Shang, Sung-Wook Hong, Aleksandra Drelich, Chien-Te K. Tseng, Marc Jenkins, Mark Heise, Lanying Du, Fang Li

**Affiliations:** 1 Department of Veterinary and Biomedical Sciences, University of Minnesota, Saint Paul, Minnesota, United States of America; 2 Center for Coronavirus Research, University of Minnesota, Saint Paul, Minnesota, United States of America; 3 Laboratory of Viral Immunology, Lindsley F. Kimball Research Institute, New York Blood Center, New York, New York, United States of America; 4 Department of Pathology and Laboratory Medicine, University of North Carolina, Chapel Hill, North Carolina, United States of America; 5 Division of Comparative Medicine, University of North Carolina, Chapel Hill, North Carolina, United States of America; 6 Department of Microbiology and Immunology, University of North Carolina, Chapel Hill, North Carolina, United States of America; 7 Rapidly Emerging Antiviral Drug Development Initiative, University of North Carolina, Chapel Hill, North Carolina, United States of America; 8 Center for Immunology, Department of Microbiology and Immunology, University of Minnesota, Minneapolis, Minnesota, United States of America; 9 Department of Microbiology and Immunology, University of Texas Medical Branch, Galveston, Texas, United States of America; 10 Department of Genetics, University of North Carolina, Chapel Hill, North Carolina, United States of America; The Peter Doherty Institute and Melbourne University, AUSTRALIA

## Abstract

The key to battling the COVID-19 pandemic and its potential aftermath is to develop a variety of vaccines that are efficacious and safe, elicit lasting immunity, and cover a range of SARS-CoV-2 variants. Recombinant viral receptor-binding domains (RBDs) are safe vaccine candidates but often have limited efficacy due to the lack of virus-like immunogen display pattern. Here we have developed a novel virus-like nanoparticle (VLP) vaccine that displays 120 copies of SARS-CoV-2 RBD on its surface. This VLP-RBD vaccine mimics virus-based vaccines in immunogen display, which boosts its efficacy, while maintaining the safety of protein-based subunit vaccines. Compared to the RBD vaccine, the VLP-RBD vaccine induced five times more neutralizing antibodies in mice that efficiently blocked SARS-CoV-2 from attaching to its host receptor and potently neutralized the cell entry of variant SARS-CoV-2 strains, SARS-CoV-1, and SARS-CoV-1-related bat coronavirus. These neutralizing immune responses induced by the VLP-RBD vaccine did not wane during the two-month study period. Furthermore, the VLP-RBD vaccine effectively protected mice from SARS-CoV-2 challenge, dramatically reducing the development of clinical signs and pathological changes in immunized mice. The VLP-RBD vaccine provides one potentially effective solution to controlling the spread of SARS-CoV-2.

## Author summary

Both mRNA-based and viral vector-based vaccines are currently being distributed to curtail the COVID-19 pandemic. Continued development of more varieties of SARS-CoV-2 vaccines will help battle the many variants of SARS-CoV-2. Here we have developed a virus-like particle (VLP) vaccine that combines the effectiveness of virus-based vaccines and safety of protein-based vaccines. Using the lumazine synthase nanoparticle protein as the structural scaffold and 120 copies of SARS-CoV-2 receptor-binding domain as the surface immunogen, this VLP vaccine induced high-titer neutralizing antibody responses in mice that lasted >2 months and potently inhibited SARS-CoV-2, SARS-CoV-1, and their variants. The VLP vaccine also protected mice from high-titer SARS-CoV-2 challenge. The novel VLP vaccine may contribute to the protection of the human population from SARS-CoV-2 and its variants.

## Introduction

Efficacious and safe vaccines are key to controlling the spread of viral infections. Thus far the FDA has approved 50 viral vaccines for clinical use to combat viral infections (https://www.fda.gov/vaccines-blood-biologics/vaccines/vaccines-licensed-use-united-states). These vaccines belong to three types: (I) 84% are virus-based vaccines (including inactivated viruses and attenuated viruses), (II) 10% are protein-based vaccines (i.e., subunit vaccines), and (III) the remaining 6% are virus-like particle vaccines (VLP vaccines). Type I, virus-based vaccines, are efficacious but may trigger safety concerns [1,2]. Attenuated virus vaccines infect people and can cause side effects; they may also revert back to wild type viruses. Inactivated virus vaccines do not infect people, but may have reduced antigenicity due to the inactivation procedure. Type II, protein-based vaccines, are usually safe because they cannot infect people. However, they may lack sufficient efficacy because the human immune system has evolved to recognize virus particles rather than individual viral proteins [3,4]. Type III, VLP vaccines, also do not infect people. They are made of protein scaffolds that present multiple copies of viral immunogens on their surface. This design maximizes the human immune responses by mimicking two unique features of virus particles: high local density of antigens and repetitive pattern of antigen display [5,6]. Thus VLP vaccines combine the advantages of virus-based and protein-based vaccines, while reducing their drawbacks [7,8].

A novel coronavirus, SARS-CoV-2, is responsible for the COVID-19 pandemic and has caused catastrophic damage to global health and economies [9,10]. A spike protein on the viral surface guides the entry of SARS-CoV-2 into host cells [11,12]. It contains a receptor-binding S1 subunit and a membrane-fusion S2 subunit. As the first step of viral entry, a defined receptor-binding domain (RBD) on SARS-CoV-2 S1 specifically binds to the cell surface receptor angiotensin-converting enzyme 2 (ACE2) for viral attachment [13–15]. Coronavirus RBDs are major immunogens on virus particles; antibodies targeting the RBDs can potentially block the viral attachment step [16,17]. We previously showed that SARS-CoV-2 RBD has significantly higher affinity for human ACE2 than does the RBD of a closely related SARS-CoV-1 [18]. Moreover, we showed that SARS-CoV-2 RBD is often hidden in the spike protein as a possible viral strategy to evade the host immune response [19]. Over the past year, many variants of SARS-CoV-2 have emerged as the virus circulated in different regions of the world. These variants contain naturally selected mutations in the spike protein including the RBD [20,21]. Thus, highly efficacious RBD-containing vaccines are needed to elicit robust immune responses to block the potent, evasive, and divergent RBD from binding to its ACE2 receptor.

Extensive efforts have been devoted to developing vaccines against SARS-CoV-2. Several nucleic acids-based vaccines (including mRNA and DNA), viral vector vaccines, and inactivated virus vaccines have reached clinical trials. As of this writing, the FDA has authorized two mRNA-based vaccines and one viral vector-based vaccine for emergency use in humans [22–24]. These vaccines have shown great promise in controlling the pandemic. However, there is a possibility that SARS-CoV-2 will become endemic; new coronaviruses may also emerge in the future. Therefore, exploration of long-term strategies is warranted. One such strategy is to develop a variety of coronavirus vaccines that complement the existing COVID-19 vaccines in combatting SARS-CoV-2 and other coronaviruses. Preclinical trials have shown that SARS-CoV-2 RBD is a candidate vaccine against SARS-CoV-2 [25]. However, over a decade of research on coronavirus RBDs indicates that the efficacy of the SARS-CoV-2 RBD vaccine will need to be further improved [26,27]. The recent development of lumazine synthase as a structural scaffold for VLP vaccines offers the promise to improve the efficacy of SARS-CoV-2 RBD vaccine [7,8]. Specifically, lumazine synthase self assembles into a 60-mer conformation, allowing the presentation of 60 copies of viral immunogens to mimic the pattern of immunogen presentation on virus particles [7,8].

In this study, we designed and developed such a VLP-based RBD vaccine and tested its immunogenicity *in vitro* and efficacy *in vivo*. Our data showed that compared to the RBD vaccine, the VLP-based RBD vaccine induced significantly higher immune responses against SARS-CoV-2, SARS-CoV-1, and their variants. Importantly, the VLP-based RBD vaccine provides highly effective protection against SARS-CoV-2 infection in a mouse model. If validated in additional preclinical models and human clinical trials, the vaccine may become a new tool in the arsenal against the spread of SARS-CoV-2.

## Results

### Design and characterization of VLP-RBD vaccine targeting SARS-CoV-2

**Fig 1:**
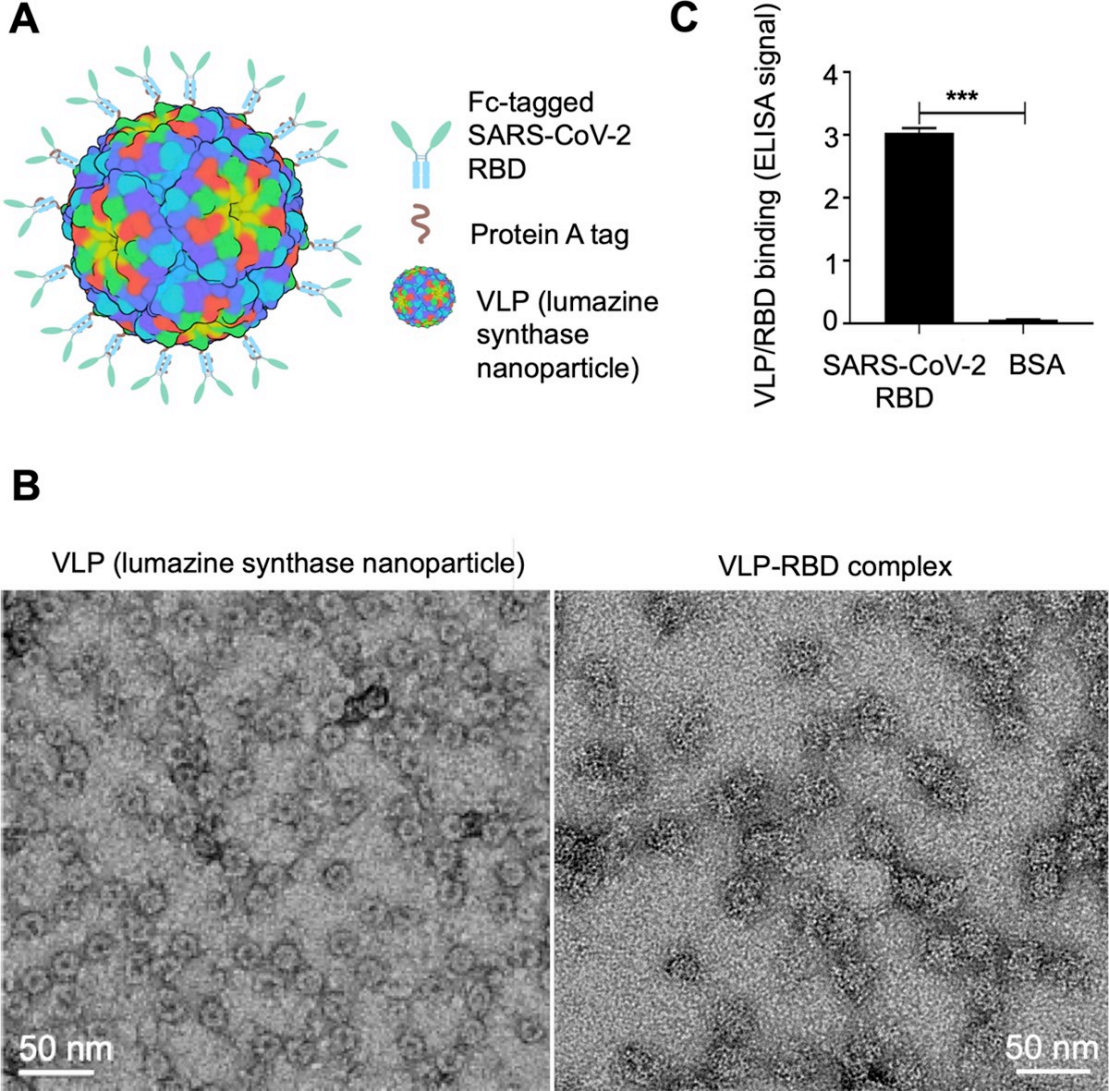
Design, construction and characterization of VLP-based SARS-CoV-2 RBD vaccine. (A) Schematic representation of Fc-tagged (light blue) SARS-CoV-2 RBD (light green), N-terminal protein A tag of nanoparticle (brown), and 60-meric VLP nanoparticle formed by bacterial lumazine synthase (colorful sphere). (B) Negative-stain EM analysis of VLP (left) and VLP-RBD complex (right). (C) Binding of VLP to SARS-CoV-2 RBD as detected using ELISA. SARS-CoV-2 RBD or equal amounts of bovine serum albumin (negative control) were coated on ELISA plate, and VLP (containing N-terminal His tag and protein A tag) was added later. Binding of VLP to RBD was detected by anti-His tag antibody. The data are presented as mean ± SEM (n = 12). A Student’s two-tailed *t*-test was performed to analyze the statistical difference between the groups. ***p < 0.001. Experiments were repeated twice with similar results.

To develop a potent VLP-based RBD vaccine, we separately constructed the lumazine synthase (containing an N-terminal protein A tag) and SARS-CoV-2 RBD (containing a C-terminal Fc tag) (Fig 1A). We expressed the lumazine synthase in both bacteria and mammalian cells and purified it to high homogeneity (S1A Fig). Independent of the expression source, the purified lumazine synthase spontaneously assembled to form a large VLP nanoparticle. The high molecular weight of the VLP nanoparticle was confirmed by its elution profile from gel filtration chromatography (S1A Fig). Further, we showed that under negative-stain electron microscopy (EM), the nanoparticle had the appearance of spheres with an average diameter of 15 nm (Fig 1B), consistent with the same 60-meric VLP nanoparticle revealed by previous EM studies [28]. We also expressed the Fc-tagged SARS-CoV-2 RBD in mammalian cells and purified it to high homogeneity (S1B Fig). The purified SARS-CoV-2 RBD formed an oligomer, likely a dimer, as evidenced by its elution profile from its gel filtration chromatography (S1B Fig). Thus, we obtained the lumazine synthase VLP nanoparticle and SARS-CoV-2 RBD both of which were well formed as expected.

Next we prepared and characterized the complex of the VLP nanoparticle and SARS-CoV-2 RBD. First, we investigated the binding interaction between the protein A-tagged VLP nanoparticle and Fc-tagged SARS-CoV-2 RBD using ELISA. The result confirmed the binding of the two proteins to each other (Fig 1C). Second, we mixed the VLP nanoparticle and SARS-CoV-2 RBD and purified the complex via gel filtration chromatography. The result revealed that the two proteins formed a tight complex in solution and were co-eluted and co-purified from gel filtration chromatography (S1C Fig). Third, we showed that under negative-stain EM, the complex of VLP nanoparticle and SARS-CoV-2 RBD (i.e., VLP-RBD) retained the spherical shape of the nanoparticle, but attained a fuzzy surface (Fig 1B), consistent with previous EM studies on the complex of the same VLP nanoparticle and a different coronavirus RBD (i.e., MERS coronavirus RBD) [28]. This design of the VLP-RBD vaccine allows 60 copies of Fc-tagged dimeric SARS-CoV-2 RBD, corresponding to 120 copies of SARS-CoV-2 RBD, to be displayed on the surface of the 60-meric protein A-tagged lumazine synthase VLP nanoparticle.

We investigated the stability of the VLP-RBD complex in the presence of competing antibodies using a protein pull-down assay. To this end, the VLP-RBD complex was incubated with mouse serum. After incubation, both free RBD molecules and VLP-associated RBD molecules were pulled down by ACE2. The VLP-RBD in the absence of mouse serum was used as a positive control where all of the VLP molecules were associated with the RBD and hence were pulled down from solution by ACE2. The VLP alone in the presence of mouse serum was used as a negative control where no VLP molecules were associated with the RBD and hence no VLP molecules were pulled down by ACE2. The result showed that compared with the two controls, the RBD molecules remained associated with the VLP after incubation with mouse serum (S1D Fig). Thus, the VLP-RBD complex remained stable in the presence of competing antibodies.

### VLP-RBD vaccine is highly potent in inducing neutralizing antibody responses in mice

**Fig 2:**
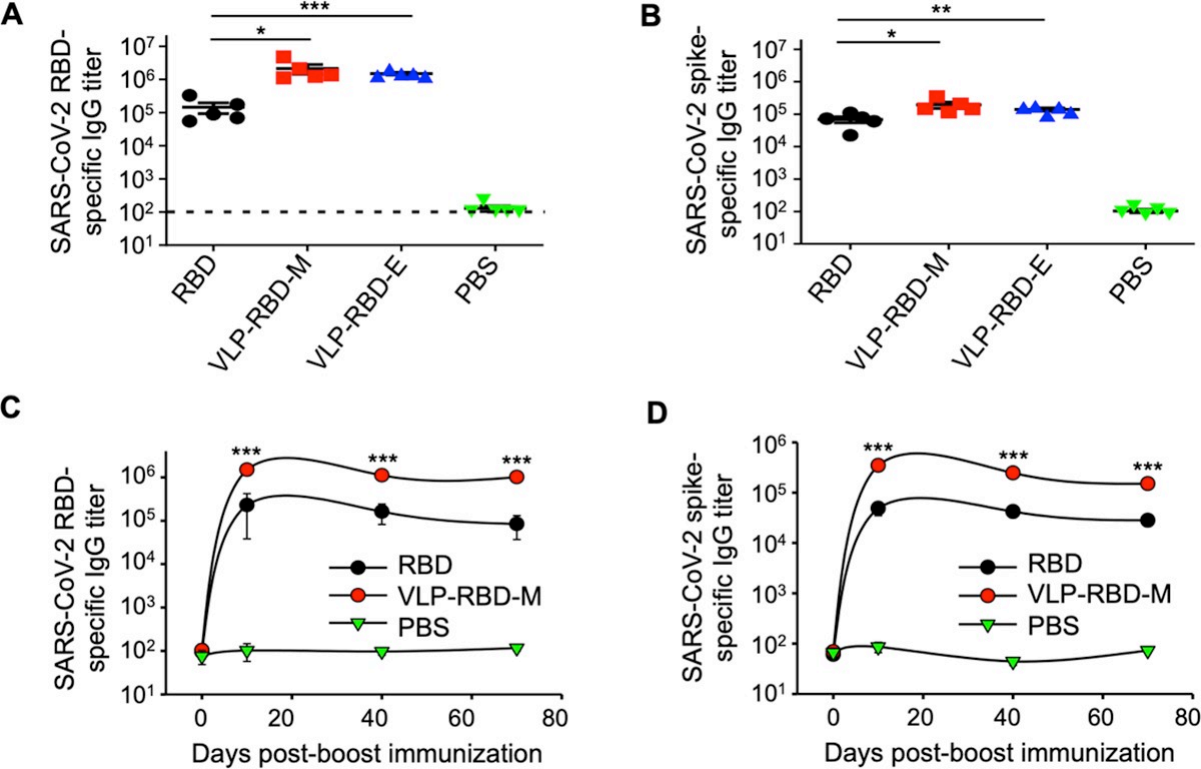
VLP-RBD vaccine induces high-titer antibody responses in mice. Mice were immunized with RBD, VLP-RBD-M (VLP was prepared from mammalian cells), VLP-RBD-E (VLP was prepared from bacterial cells), or PBS. Mouse sera were collected on day 10 post-2^nd^ immunization for detection of RBD-specific antibodies (A) and spike-specific IgG antibodies (B) using ELISA. Mouse sera were also collected on later dates for detection of RBD-specific antibodies (C) and spike-specific IgG antibodies (D). For ELISA, plates were pre-coated with recombinant SARS-CoV-2 RBD or spike ectodomain, and antibody titers were reported as the highest serum dilution that remained detectable (defined as signal being at least twice of the blank). The dotted line indicates the detection limit (defined as the lowest serum dilution when signal was still below detectable levels). The data are presented as mean ± SEM (n = 5 for each mouse group). A Student’s two-tailed *t*-test was performed to analyze the statistical differences among the groups. ***p < 0.001; **p < 0.01; *p < 0.05. Experiments were repeated twice with similar results.

**Fig 3:**
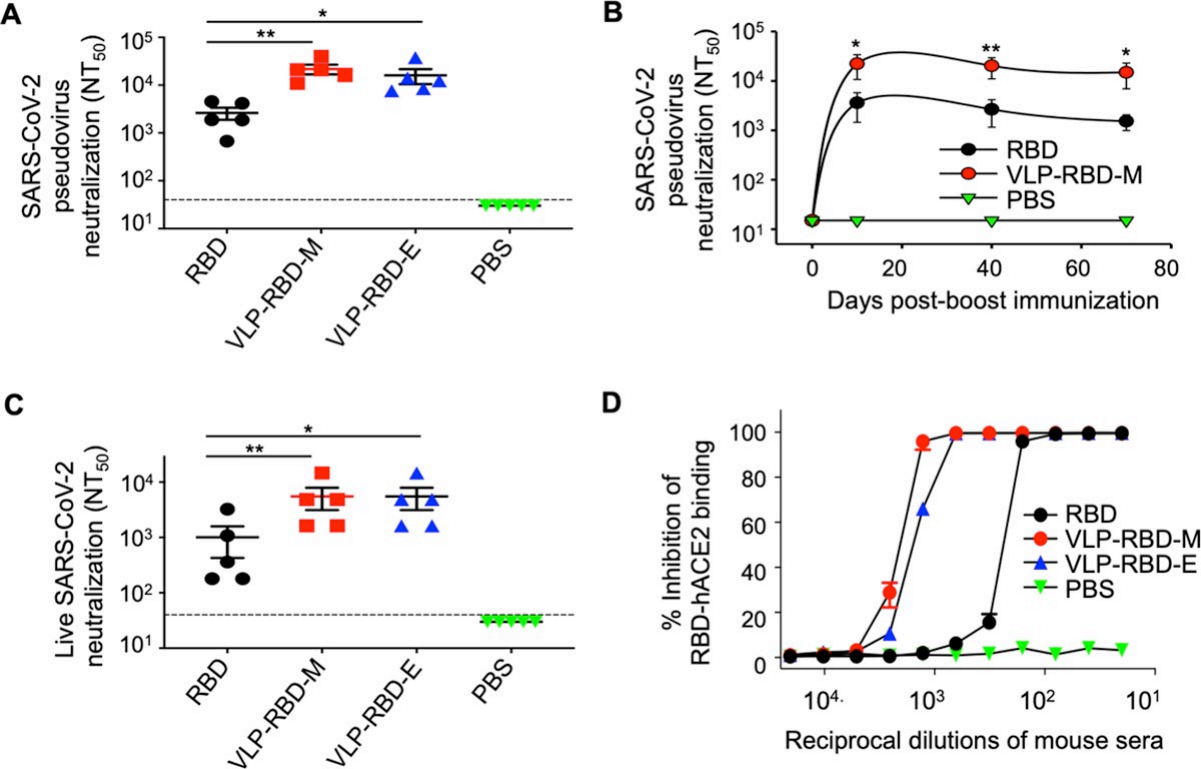
Antibody responses induced by VLP-RBD vaccine potently neutralize SARS-CoV-2 infection *in vitro*. Mouse sera from day 10 post-2^nd^ immunization were examined for neutralizing antibodies against cell entry of pseudotyped SARS-CoV-2 (A) and cell infection of authentic SARS-CoV-2 (C). Mouse sera collected on later dates were also examined for neutralizing antibodies against cell entry of pseudotyped SARS-CoV-2 (B). For pseudovirus entry assay, retroviruses pseudotyped with SARS-CoV-2 spike were used to enter human cells in the presence of serially diluted mouse sera, and efficiency of pseudovirus entry was characterized as luciferase signal accompanying entry. For live virus assay, live SARS-CoV-2 was used to infect human cells in the presence of serially diluted mouse sera, and efficiency of live virus infection was characterized as cytopathic effect (CPE) of infected target cells. Neutralizing activity of serum antibodies against pseudoviruses or live SARS-CoV-2 was expressed as NT_50_ (neutralization titer that inhibits pseudovirus entry or live virus infection by 50%). The dotted lines indicate the detection limit for each experiment (defined as the lowest serum dilution). (D) Mouse sera from day 10 post-2^nd^ immunization were tested for blocking the interaction between SARS-CoV-2 RBD and human ACE2 receptor using flow cytometry. Recombinant RBD was incubated with cells expressing ACE2 in the presence of mouse sera, and efficiency of binding was characterized by flow cytometry signal (i.e., fluorescence intensity of cells). Inhibition (%) was derived from flow cytometry signal in the presence or absence of mouse sera. The data are presented as mean ± SEM (n = 5 for mice in each group). A Student’s two-tailed *t*-test was performed to analyze the statistical differences among the groups. ***p < 0.001; **p < 0.01; *p < 0.05. Experiments were repeated twice with similar results.

We evaluated the potency of the VLP-RBD vaccine in inducing neutralizing antibody responses against SARS-CoV-2. To this end, we immunized mice with one of two types of the VLP-RBD vaccines: VLP-RBD-E with the nanoparticle protein expressed from bacteria and VLP-RBD-M with the nanoparticle protein expressed from mammalian cells. The Fc-tagged RBD was used for comparison. The mice were further boosted with the same immunogen at 4 weeks after the prime immunization. Mouse sera were collected on days 10, 40, and 70 after the second immunization, and analyzed for their antibody titers and potency in neutralizing the cell entry of SARS-CoV-2. First, compared to the RBD vaccine, the mouse sera induced by the VLP-RBD vaccine contained >5 times more RBD-specific and spike-specific IgG antibodies, respectively (Fig 2A and 2B); they also neutralized the cell entry of SARS-CoV-2 pseudoviruses more potently (Fig 3A). Second, compared to day 10, the mouse sera induced by the VLP-RBD vaccine and collected on days 40 and 70 contained similar amounts of RBD-specific and spike-specific IgG antibodies (Fig 2C and 2D) and neutralized the cell entry of SARS-CoV-2 pseudoviruses with similar potency (Fig 3B). Third, compared with the RBD vaccine, the mouse sera induced by the VLP-RBD vaccine neutralized the cell infection of authentic SARS-CoV-2 more potently (Fig 3C). Fourth, VLP-RBD-E and VLP-RBD-M showed comparable potency in inducing neutralizing antibody titers (Figs 2A, 2B, 3A and 3C) as well as in other parameters (see below), revealing that the nanoparticles prepared from mammalian cells and bacteria performed similarly well as the structural scaffold for the VLP-RBD vaccine. Finally, compared to the second immunization, the vaccine-induced mouse sera collected after the prime immunization contained significantly lower levels of RBD-specific IgG antibodies (Figs 2A and S2A) and neutralized the cell entry of SARS-CoV-2 pseudoviruses less well (S2B Fig); moreover, like the PBS buffer, VLP alone did not induce RBD-specific IgG antibodies (S2A Fig). Together, these results demonstrate that compared to the RBD vaccine, the VLP-RBD vaccines induce higher-titer neutralizing antibody responses and inhibit SARS-CoV-2 infection more potently; additionally, these neutralizing antibody responses were boosted by the second immunization and last relatively long term.

**Fig 4:**
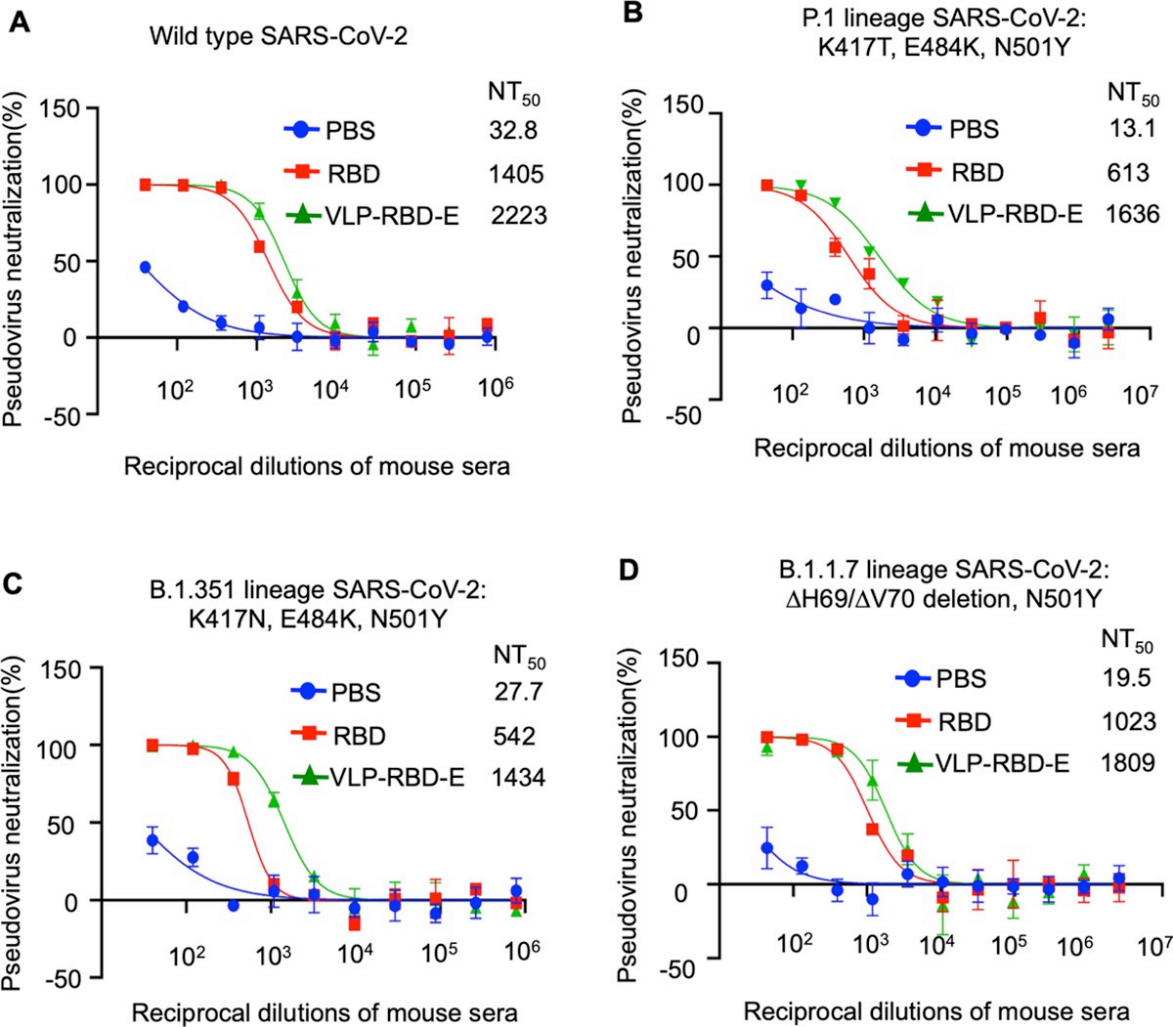
Antibody responses induced by VLP-RBD vaccine potently neutralize the cell entry of pseudotyped SARS-CoV-2 variants. The experiments were performed in the same way as in Fig 3A, except that a different pseudovirus system was used (see Materials and Methods) and also the mouse sera in each immunization group were pooled together. The mutations in the S1 subunit of the SARS-CoV-2 variants (each defined as a lineage) are listed. Except for the ΔH69/ ΔH70 deletions, all of the other mutations are located in the RBD.

Next we examined whether the antibody responses induced by the VLP-RBD vaccine neutralize different SARS-CoV-2 variants and other related coronaviruses. During the progression of the COVID-19 pandemic, many SARS-CoV-2 variants have emerged. Among them, the recently emerged variants of P.1 lineage, B.1.351 lineage, and B.1.1.7 lineage are of great concern, owing to their ability to escape immune surveillance, spread more efficiently, and cause more severe disease [20,21]. These variants contain mutations in the S1 subunit, particularly in the RBD [20,21]. We generated SARS-CoV-2 pseudoviruses corresponding to each of these three SARS-CoV-2 variants. We showed that compared to the wild type SARS-CoV-2 pseudoviruses, the mouse sera induced by the VLP-RBD vaccine neutralized the cell entry of the SARS-CoV-2 variant pseudoviruses with similar potency (Fig 4A–4D). Additionally, compared to the SARS-CoV-2 variants, the wild type SARS-CoV-2 spike protein shares lower but still significant sequence similarities with the spike proteins from SARS-CoV-1 and SARS-CoV-1-like bat coronaviruses (e.g., bat coronavirus SHC014) [13,29]. Compared to the SARS-CoV-2 pseudoviruses, the mouse sera induced by the VLP-RBD vaccine neutralized the cell entry of both SARS-CoV-1 and SHC014 pseudoviruses with lower but still significant potency (Figs 3A, S3A and S3B). Overall, these data demonstrate that the VLP-RBD vaccine elicited antibody responses with potent cross-neutralizing capabilities against SARS-CoV-2 variants and significant cross-neutralizing capabilities against SARS-CoV-1 and its related bat coronavirus.

To investigate the mechanism by which the vaccine-induced antibodies neutralize SARS-CoV-2 infection, we examined the interactions between SARS-CoV-2 RBD and human ACE2 in the presence of the vaccine-induced mouse sera. To this end, we performed a flow cytometry assay where recombinant SARS-CoV-2 RBD was incubated with cell-surface-expressed human ACE2 in the presence of mouse sera. The result showed that mouse antibodies induced by either the RBD vaccine or VLP-RBD vaccine strongly blocked SARS-CoV-2 RBD binding to human ACE2, and that the latter sera were more potent than the former (Figs 3D and S4). These data reveal that compared to the RBD vaccine, the VLP-RBD vaccine induces significantly higher-titer antibodies that block SARS-CoV-2 RBD binding to human ACE2 and neutralize SARS-CoV-2 infection of target cells.

### VLP-RBD vaccine effectively protected mice from SARS-CoV-2 challenge

**Fig 5:**
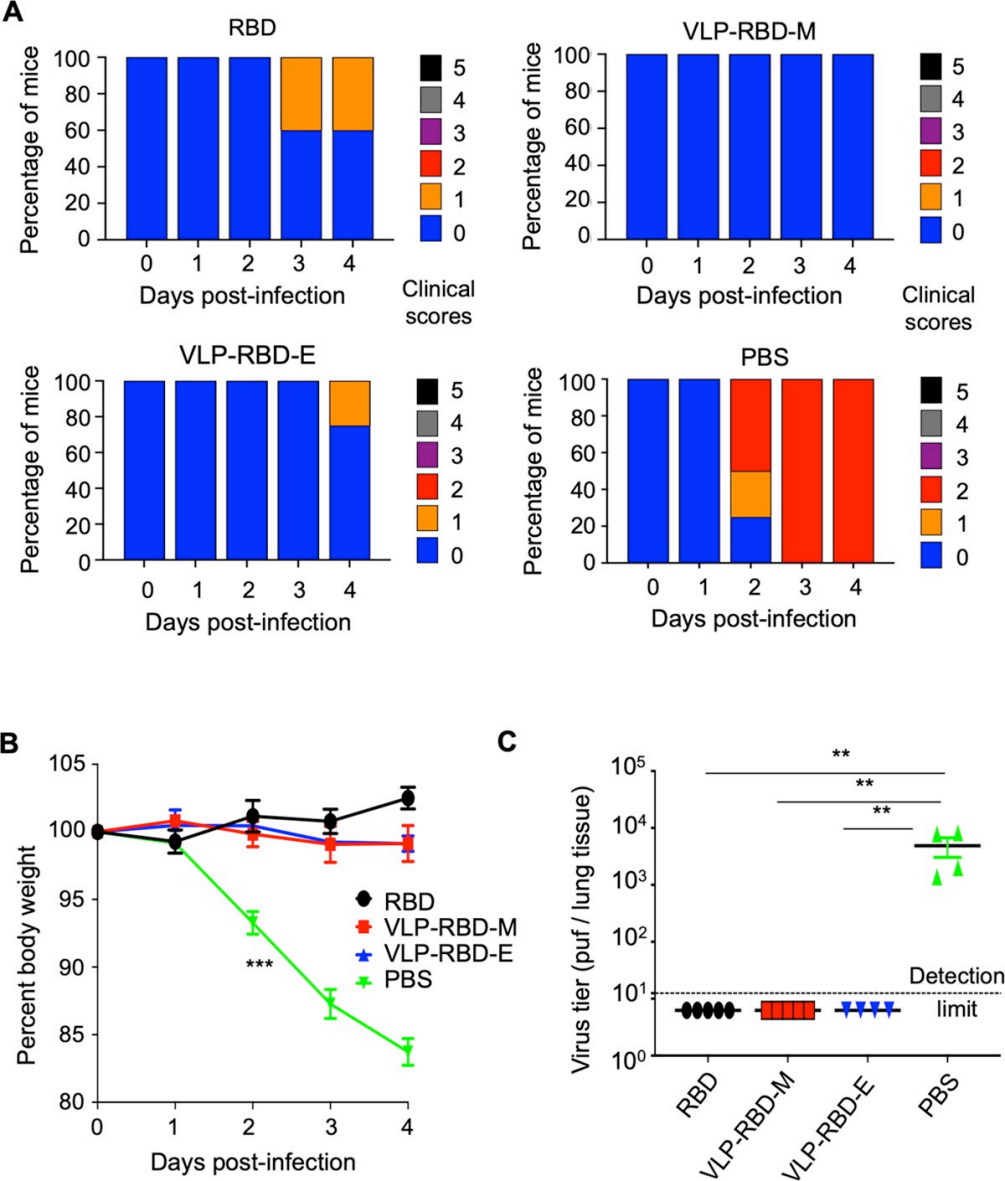
VLP-RBD vaccine protects mice from SARS-CoV-2 infection *in vivo*. Mice were immunized with VLP-RBD vaccine, RBD vaccine or PBS, and were then intranasally challenged with a mouse-adapted SARS-CoV-2 strain. Clinical scores (A), body weights (B), and virus titers in the lung tissue (C) of mice were recorded. The data are presented as mean ± SEM (n = 4–5 for mice in each group). For (B), a two-way ANOVA with a Dunnett’s multiple comparisons post-test was performed to analyze the statistical differences among the groups. For (C), a Kruskal-Wallis test with Dunn’s multiple comparisons was performed to analyze the statistical differences among the groups. ***p < 0.001; **p < 0.01.

We investigated the efficacy of the VLP-RBD vaccine in protecting animal models against SARS-CoV-2 infection. To this end, 3 weeks after the second immunization with either the RBD vaccine or VLP-RBD vaccine (adjuvants were also included), the immunized mice were challenged with a mouse-adapted SARS-CoV-2 strain at high dosage (10^5^ pfu) [30]. This mouse model of pathogenic SARS-CoV-2 infection replicates many of the features seen in severe COVID-19 cases in humans, including acute respiratory distress syndrome and acute lung injury [30]. Here clinical disease was assessed by evaluating mice for clinical scores and weight changes, viral lung load was measured by plaque assay for infectious virus, and lung pathology was evaluated by scoring tissue for histopathological acute lung injury (ALI), gross lung discoloration (GLD) and diffuse alveolar damage (DAD) [31–33]. Sham vaccinated mice immunized with PBS and adjuvants (referred to as PBS thereafter) were used as negative controls. On day 2, the sham vaccinated mice began to show progression in clinical scores and weight loss (Fig 5A and 5B). By day 4, the sham vaccinated mice had lost an average of ~15% of their starting body weight and all of them had progressed to a clinical score of 2 (Fig 5A and 5B). On day 4, the sham vaccinated mice contained high virus titers in the lung tissue (mean titer: 4.9 x 10^3^ pfu per lung tissue) (Fig 5C) and developed gross and histopathological signs of significant lung injury (average scores: 1 for GLD; 0.52 for ALI; 2.9 for DAD) (S5 Fig). In contrast, mice immunized with either the RBD vaccine or the VLP-RBD vaccine showed no significant weight loss, contained no detectable infectious virus in the lung tissue, and developed minimal pathological changes of the lung (average scores: 0.2 for GLD; ~0.2 for ALI; ~1.1 for DAD) on day 4 (Figs 5B, 5C and S5). However, among the five mice immunized with the RBD vaccine, two mice developed clinical signs scored at 1 on days 3 and 4 and one developed a GLD score of 1 on day 4 (Figs 5A and S5A). In comparison, among the four mice immunized with the VLP-RBD-E vaccine, none developed clinical signs or any GLD on day 4; among the five mice immunized with the VLP-RBD-M vaccine, none developed clinical signs, and one developed a GLD score of 1 on day 4 (Figs 5A and S5A). Overall, these results demonstrate that the VLP-RBD vaccine offers nearly complete protection for mice against SARS-CoV-2 infection.

## Discussion

Coronavirus vaccines hold the key to ending the COVID-19 pandemic and fighting potential future coronavirus infections. Currently SARS-CoV-2 vaccines, including mRNA vaccines, vector-based vaccines and inactivated virus particles, have been entering human clinical trials at an unprecedented speed. Among these vaccines, two mRNA-based vaccines and one viral vector-based vaccine have received FDA authorization for emergency use in humans. The demand for vaccines, however, far exceeds the supply. Together with an increase of SARS-CoV-2 variants, there is a pressing need for an increase in the variety of vaccines against SARS-CoV-2 and potential future coronavirus infections. Among the FDA-approved viral vaccines, 10% are protein-based subunit vaccines. Subunit vaccines do not contain any infectious viral components and hence are considered safer than virus-based vaccines. However, some subunit vaccines have relatively low immunogenicity. Coronavirus spike protein RBDs, which attach coronaviruses to host cells as the first step of infection, are one of the most immunogenic components among all coronavirus proteins, and hence they are the prime targets for subunit vaccine design. We previously used a glycan shield approach to enhance the neutralizing immunogenicity of coronavirus RBDs as subunit vaccines [26]. In this study, we used a virus-like nanoparticle approach to enhance the neutralizing immunogenicity of SARS-CoV-2 RBD as a subunit vaccine. Specifically, we prepared a nanoparticle scaffold protein that spontaneously assembled into a 60-mer VLP, displaying 60 copies of N-terminal protein A tag on the surface; we also prepared Fc-tagged dimeric SARS-CoV-2 RBD. When the protein A-tagged VLP nanoparticle and the Fc-tagged RBD were conjugated together, the assembled nanoparticle scaffold was capable of presenting 120 copies of RBD on the surface. The high local density and repetitive pattern of the presented RBD on the surface of the assembled VLP nanoparticle mimic those on the virus particles, likely accounting for the VLP-RBD vaccine’s ability to induce potent and long-lasting immune responses [5,6]. Therefore, our design of the VLP-based SARS-CoV-2 RBD vaccine holds promise for a novel SARS-CoV-2 vaccine with enhanced neutralizing immunogenicity.

We also extensively examined the immunogenicity and protective efficacy of the VLP-RBD vaccine using the RBD vaccine as a comparison. Our study showed that both the VLP-RBD vaccine and the RBD vaccine trigger robust antibody responses in mice that potently block SARS-CoV-2 binding to its ACE2 receptor and neutralize SARS-CoV-2 infection of human cells. In addition, the VLP-RBD vaccine is about five times more potent than the RBD vaccine in triggering neutralizing antibody responses in mice. The immune responses triggered by these vaccines lasted at least two months during the detection period. Moreover, both the VLP-RBD vaccine and the RBD vaccine effectively protected mice from challenge with a mouse-adapted SARS-CoV-2 strain, as evidenced by no significant weight loss, low or absent clinical scores, undetectable virus titer in the lungs and decreased lung pathology of vaccinated mice. However, whereas a subset of the mice immunized with the RBD vaccine still exhibited low disease scores, immunization with the VLP-RBD vaccine provided nearly complete protection from clinical disease, which is consistent with the observation that the VLP-RBD vaccine triggers much stronger neutralizing antibody responses than the RBD vaccine. It is also worth noting that the VLP-RBD vaccine works not only against wild type SARS-CoV-2, but also against SARS-CoV-2 variants, the related SARS-CoV-1, and a SARS-CoV-1-like bat coronavirus. Our data suggest that these viruses share many neutralizing epitopes on their structurally related RBDs and that the VLP-RBD vaccine triggers potent immune responses to target these conserved neutralizing epitopes. Thus, our VLP-RBD vaccine has the potential to be effective against a variety of SARS-CoV-2 strains, easing concerns about potential vaccine-escaping SARS-CoV-2 mutations. Overall, our novel design of the VLP-based SARS-CoV-2 RBD vaccine is highly potent in triggering robust and long-term immune responses against SARS-CoV-2 as well as its variants and in protecting animals from SARS-CoV-2 challenge.

In addition to their potency, cost-effectiveness is an important factor to consider for SARS-CoV-2 vaccines, given the world’s vast population and the potential emergence of other coronaviruses. In this study, we prepared the VLP nanoparticle from both bacteria and mammalian cells, and showed that they performed similarly well as the structural scaffold for the VLP-RBD vaccine. This result is significant because it is more convenient and cost-effective to prepare proteins from bacteria than from mammalian cells. Although we prepared Fc-tagged SARS-CoV-2 RBD from mammalian cells to preserve the immunogenicity of the RBD, the yield of the RBD from mammalian cells was high due to the relatively small size and good folding of the RBD. Therefore, our design of the VLP-RBD vaccine enhances cost-effectiveness.

Given the fast evolution of SARS-CoV-2 and potential future emergence of novel coronaviruses, versatility in vaccine design may allow quick development of new vaccines to battle future coronavirus infections. The separate preparations of the nanoparticle and the RBD and then conjugating the two components together through tags afford versatility to the vaccine. This approach is important if the SARS-CoV-2 RBD component needs additional engineering, if another SARS-CoV-2 immunogen is incorporated into the VLP system, if a completely different coronavirus RBD vaccine is needed, or if the conjugation tags need to be modified. For example, full-length coronavirus spike ectodomains have many advantages over the RBDs as coronavirus vaccines due to their large size and many useful non-RBD epitopes [17]. However, recombinant coronavirus spike ectodomains typically give low production yields due to their large size and relatively unstable folding, making cost-effectiveness a potential obstacle for large-scale productions. Nevertheless, as a versatile system, the VLP system may be used to present the SARS-CoV-2 spike ectodomain, yielding a VLP-spike vaccine with superior efficacy. Overall, the VLP nanoparticle vaccine system allows changes of immunogens and modifications of conjugation tags for further performance improvement and future clinical needs.

Though widely regarded as a successful bioengineering strategy [34], the Fc fusion technology has two potential drawbacks. First, although many Fc-fusion therapeutics have been clinically approved for human use, it has been shown that the Fc tag in these therapeutics may modulate immunogenicity *in vivo* by binding to the Fc receptors in some immune cells and activating these cells [35]. Second, although very strong, the noncovalent interaction between the Fc and protein A tags [36] could lead to gradual displacement of the Fc-tagged RBD by IgG antibodies *in vivo*, potentially decreasing the potency of the VLP-RBD vaccine. These potential complications can be avoided by replacing the pair of Fc and protein A tags with either a pair of noncovalent tags that do not involve Fc or a pair of tags that form covalent interactions upon binding each other [37–39]. It is important to note, however, that subunit vaccines are not administered intravenously, an approach that can dilute any subunit vaccines and lead to their enzymatical degradation. Instead, intramuscular injection allows subunit vaccines to stay concentrated and stable inside muscle tissues, allowing for uptake, processing and presentation by local antigen presenting cells (APCs). Subsequently these APCs move to nearby lymph nodes to activate immune cells [40]. Our data showed that the VLP-RBD complex is stable in the presence of competing antibodies in mouse serum (S1D Fig). The stability of the VLP-RBD vaccine, when administered intramuscularly, is likely much higher. Importantly, our data demonstrated that the VLP-RBD vaccine is more potent than the RBD vaccine in inducing neutralizing antibodies in mice.

During the preparation of this manuscript, another study on the nanoparticle-based SARS-CoV-2 RBD vaccine was published where a computationally designed nanoparticle was used to present 60 copies of the RBD [41]. Our VLP-RBD vaccine uses the lumazine synthase as the structural scaffold, which presents 120 copies of the RBD. Moreover, this study uniquely showed that our VLP-RBD vaccine triggers long-term neutralizing immune responses in mice and confers nearly complete protection to mice against high-dose SARS-CoV-2 infection. Our VLP-RBD vaccine also demonstrates a broad-spectrum activity against SARS-CoV-2 variants and other related coronaviruses with pandemic potential. Both studies support each other in the concept of using nanoparticles as structural scaffolds to present the SARS-CoV-2 RBD immunogen.

To summarize, we have designed and produced a novel VLP-based RBD vaccine targeting SARS-CoV-2. We have demonstrated its potency in inducing strong and long lasting neutralizing immune responses against not only SARS-CoV-2 and its variants but also SARS-CoV-1 and its related bat coronavirus. We have also shown that the vaccine provides nearly complete protection against SARS-CoV-2 infection in available animal models. The preparation of this vaccine is easy, cost-effective and versatile. If further validated in additional preclinical models and human clinical trials, this vaccine can complement the existing vaccines in controlling the spread of SARS-CoV-2.

## Materials and methods

### Ethics statement

All animal work was performed in strict accordance with the guidance and recommendations in the Guide for the Care and Use of Laboratory Animals (National Research Council Institute for Laboratory Animal Research). Experiments were conducted under animal use protocols approved by the Institutional Animal Care and Use Committees at the New York Blood Center and at the University of North Carolina at Chapel Hill.

All live SARS-CoV-2 work was performed under BSL3 conditions at the University of Texas Medical Branch and the University of North Carolina at Chapel Hill, following all safety precautions recommended by *Biosafety in Microbiological and Biomedical Laboratories* (BMBL).

### Cell lines and plasmids

HEK293T cells (human embryonic kidney cells) were obtained from the American Type Culture Collection (ATCC) and cultured in Dulbecco’s modified Eagle medium (supplemented with 10% fetal bovine serum, 2 mM L-glutamine, 100 units/mL penicillin, and 100 μg/mL streptomycin). FreeStyle 293-F Cells were purchased from Gibco and cultured in FreeStyle 293 Expression Medium (Gibco) (supplemented with 100 units/mL penicillin and 100 μg/mL streptomycin). BL21 (DE3) *E*. *coli* cells were obtained from New England Biolabs and cultured in Luria Broth (Tryptone: 10 g/L, Yeast: 5 g/L, Sodium Chloride: 10 g/L) and 50 μg/mL Kanamycin.

Genes encoding SARS-CoV-2 spike (GenBank accession number QHD43416.1), SARS-CoV-1 spike (GenBank accession number AFR58740.1), SARS-CoV-1-related bat SHC014 spike (GenBank accession number AGZ48806.1), human ACE2 (GenBank accession number NM_021804), lumazine synthase of the hyperthermophile *Aquifex aeolicus* bacterium (GenBank accession number WP_010880027.1), and domain B of protein A from *S*. *aureus* (UniProt accession number P38507) were all synthesized (GenScript). SARS-CoV-2 RBD (residues 319–535) or SARS-CoV-1 RBD (residues 306–515) was subcloned into pLenti-transfer vector (Addgene) with an N-terminal tissue plasminogen activator (tPA) signal peptide and a C-terminal human IgG4 Fc region. Fc fragment was constructed in the same way as SARS-CoV-2 RBD, except that the RBD region was absent. Lumazine synthase was subcloned into pET-42 b(+) vector (Addgene) with an N-terminal His_8_-tag and a domain B of protein A (residues 212–270). ACE2 ectodomain (residues 1–615) was subcloned into pLenti-transfer vector with an N-terminal TPA signal peptide and a C-terminal His_8_-tag. The plasmids of 2^nd^ generation lentiviral packaging plasmid (psPAX2) (Addgene) and VSV-G envelope expressing plasmid (pMD2.G) (Addgene) were used for packaging lentiviral particles used for stable cell construction. The pLKO.1 Protocol from Addgene was followed to generate the lentiviral particles. Puromycin (Gibco) was used for the selection of stable cell lines.

### Protein expression and purification

Proteins were expressed from 293F cells as previously described [42]. Briefly, the Fc-tagged proteins (SARS-CoV-2 RBD and Fc fragment alone) were collected from the cell culture medium, purified using Protein A column and gel filtration column (Cytiva). Mammalian cell-expressed and His-tagged lumazine synthase VLP nanoparticle protein (i.e., VLP-M) was prepared in the same way as the Fc-tagged SARS-CoV-2 RBD, except that Ni-NTA column replaced the protein A column in the procedure. *E*. *coli*-expressed and His-tagged lumazine synthase VLP nanoparticle protein (i.e., VLP-E) was expressed from BL21 (DE3) *E*. *coli* cells. Protein expression was induced using isopropyl-beta-D-thiogalactoside (Santa Cruz Biotechnology) at a final concentration of 1 mM when bacterial growth reached the logarithmic phase. After induction, bacteria were allowed to grow for 4–5 h at 37°C (shake speed 250 rpm). VLP-E was harvested from bacterial cytoplasm and purified in the same way as VLP-M. To prepare VLP-RBD, the purified VLP nanoparticle protein was incubated with Fc-tagged SARS-CoV-2 (the latter was in molar excess) at room temperature for 1 h and subsequently the formed complex was purified using gel filtration chromatography. Recombinant SARS-CoV-2 spike ectodomain was purchased from Sino Biological Inc.

### Protein pull-down assay

Protein pull-down assay was performed using an EZ-Link Sulfo-NHS-LC-Biotinylation Kit (Pierce Biotechnology) according to the manufacturers’ manual. Briefly, recombinant human ACE2 (containing a C-terminal His tag) was biotinylated through incubation with Sulfo-NHS-LC-Biotin at room temperature for 1 h. Excess non-reacted and hydrolyzed biotin was removed through a desalting column provided by the kit. In the meanwhile, 200 pmol VLP-RBD was pre-incubated with 100 μl mouse serum at room temperature for 2 h. As two controls, 200 pmol VLP (containing N-terminal protein A tag and His tag) was pre-incubated with 100 μl mouse serum, whereas 200 pmol VLP-RBD was pre-incubated with 100 μl PBS buffer. Then each of the above groups (one experimental group and two control groups) was incubated with 10 μg biotinylated ACE2 for 1 h. Subsequently, each of the groups was incubated with 50 μl Streptavidin Magnetic Beads (10 mg/ml) on a roller at room temperature for 1 h. After the beads were washed three times with PBST buffer (PBS buffer + 0.1% Tween-20), 100 μl SDS-PAGE reducing sample buffer (4x premixed Laemmli protein sample buffer containing 10% β-mercaptoethanol) was added and the samples were heated at 96–100°C in a heating block for 5 min. The beads were magnetically removed and the supernatants in each group were subjected to SDS-PAGE and analyzed by Western blotting using an anti-His tag antibody (Santa Cruz Biotechnology).

### Negative staining analysis

Negative-stain electron microscopy was performed as previously described [43]. Briefly, the VLP-RBD-E sample was diluted to a final concentration of 0.3 mg/mL in Tris-HCl buffer (pH 10.0) and 200 mM NaCl, and was then loaded onto glow-discharged 400-mesh carbon grids (Electron Microscopy Sciences). The grids were stained with 0.75% uranyl formate. All micrographs were acquired using a FEI Tecnai G^2^ F30 at 120 keV (FEI Company) and a 4k x 4k Ultrascan CCD camera at 80,000× magnification at the University of Minnesota.

### Immunization of mice

Female BALB/cJ mice (6–8 weeks old) (Jackson labs, stock 000651) were intramuscularly (I.M.) immunized with recombinant Fc-tagged SARS-CoV-2 RBD (10 μg/mouse), VLP-RBD-E (10 μg/mouse), VLP-RBD-M (10 μg/mouse), VLP-E alone (10 μg/mouse), or PBS buffer in the presence of two adjuvants: aluminum hydroxide (Alum, 500 μg/mouse; InvivoGen) and monophosphoryl lipid A (MPL, 10 μg/mouse; InvivoGen). The mice were further boosted with same immunogen via I.M. at 4 weeks. Two separate experimental procedures were then performed. (i) Mouse sera were collected on day 10 post-1^st^ immunization, right before the 2^nd^ immunization, and on days 10, 40, and 70 post-2^nd^ immunization and were used for detection of antibody responses. (ii) 3 weeks post-2^nd^ immunization, mice were challenged with mouse-adapted SARS-CoV-2 (see below).

### ELISA

ELISA was carried out to detect the interaction between SARS-CoV-2 RBD and the VLP nanoparticle as previously described [42]. Briefly, ELISA plates were coated by 500 ng SARS-CoV-2 RBD (containing a C-terminal Fc tag) or equal amount of bovine serum albumin (which served as the negative control) at 4°C overnight, and then washed with wash buffer (PBS + 0.1% Tween-20). Subsequently, the ELISA plates were blocked with 5% milk at room temperature for 1 h. Then 250 ng VLP nanoparticle protein (containing N-terminal His tag and protein A tag) was added. After 2 h incubation at room temperature, the ELISA plates were washed and anti-His monoclonal antibody (1:1000) (Santa Cruz Biotechnology) was added. After another 1 h incubation at room temperature and more washes, secondary horseradish peroxidase (HRP)-conjugated anti-mouse antibodies (1:1000) (Santa Cruz Biotechnology) was added. After another 1 h incubation at room temperature, ELISA substrate (Sigma-Aldrich) was added. The ELISA reaction was stopped using 1N H_2_SO_4_. The ELISA signal was read using the Epoch Microplate Spectrophotometer (BioTek Instruments) at the 450 nm wavelength.

ELISA was also performed to detect the interaction between SARS-CoV-2 RBD and RBD-specific antibodies in mouse sera as well as the interaction between SARS-CoV-2 spike ectodomain and spike-specific antibodies in mouse sera. The procedure was the same as described above, except that the ELISA plates were coated with 50 ng RBD or spike ectodomain and then sequentially incubated with serially diluted mouse sera (instead of VLP nanoparticle protein) and HRP-conjugated anti-mouse antibodies (1:5000) (Thermo Fisher Scientific).

### Pseudovirus neutralization assay

The mouse sera collected above were examined for neutralizing antibodies against the cell entry of pseudotyped SARS-CoV-2, SARS-CoV-2 variants, SARS-CoV-1, and SARS-CoV-1-related bat coronavirus SHC014. Two types of pseudovirus systems were used. First, the pseudovirus particles for SARS-CoV-2, SARS-CoV-1, and SHC014 (Figs 3A, 3B and S3B) were packaged as previously described [42]. Briefly, HEK293T cells were co-transfected with a plasmid encoding one of the coronavirus spike proteins and a plasmid encoding Env-defective, luciferase-expressing HIV-1 genome (pNL4-3.luc.RE). Second, the pseudovirus particles for SARS-CoV-2 and its variants (Figs 4A–4D, and S2B) were packaged as previously described [44]. Briefly, HEK293T cells were co-transfected with a plasmid encoding one of the coronavirus spike proteins, a helper plasmid psPAX2 and a reporter plasmid plenti-CMV-luc. For both pseudovirus systems, pseudoviruses were collected from culture supernatants at 72 h post-transfection and were incubated with serially diluted mouse sera at 37°C for 1 h. The mixtures were then added to HEK293T cells stably expressing human ACE2 [42]. After incubation at 37°C for 72 h, the cells were lysed and transferred to luminometer plates. Then the luciferase substrate (Promega) was added and the relative luciferase activity was measured using Infinite 200 PRO Luminometer (Tecan). Neutralizing activity of serum antibodies against pseudoviruses was expressed as NT_50_ (neutralization titer that inhibits pseudovirus entry by 50%).

### Live virus neutralization assay

The mouse sera collected above were examined for neutralizing antibodies against cell infection of live SARS-CoV-2 as previously described [45]. Briefly, serially diluted mouse sera were mixed with SARS-CoV-2 (isolate US-WA-1; ~120 median tissue culture infectious dose or TCID_50_), and incubated at room temperature for 1 h. The mixtures were subsequently added to Vero E6 cells pre-plated in 96-well tissue culture plates. The cells were then cultured at 37°C for three days. Cells with or without virus were used as positive or negative control, respectively. Cytopathic effect (CPE) of cells was recorded on day 3 post-infection. Neutralizing antibody titer was expressed as the highest dilution of mouse sera being able to completely prevent virus-caused CPE in at least 50% of the wells.

### Flow cytometry

Flow cytometry was performed to detect the interaction between the SARS-CoV-2 RBD and ACE2 in the presence of mouse sera. Briefly, human ACE2-expressing HEK293T cells were incubated with recombinant Fc-tagged SARS-CoV-2 RBD (0.1 μg/ml) in the presence or absence of serially diluted mouse sera at room temperature for 1 h. After three washes with PBS (containing 2% FBS), the cells were incubated with FITC-labeled goat anti-human IgG-Fc antibody (1:500) (Sigma-Aldrich) at room temperature for 20 min. After more washes, the cells were fixed with 4% formaldehyde and the fluorescence intensity of the cells was measured using flow cytometry (BD LSRFortessa 4 system).

### Mouse infections and tissue collection

Mice were anesthetized with a cocktail of 50 mg/kg ketamine and 15 mg/kg xylazine and intranasally inoculated with 10^5^ plaque-forming unit (pfu) SARS-CoV-2 (mouse-passaged MA10; diluted in 50 μL PBS) [30]. Body weights of mice were measured daily, and clinical disease was assessed using a 0-5-point scoring system: 0 = normal; 1 = piloerection, 2 = piloerection and kyphosis; 3 = piloerection, kyphosis, and reduced movement; 4 = piloerection, kyphosis, minimal spontaneous movement, +/- labored breathing (humane endpoint); 5 = moribund, dead, or euthanized. Researchers were blinded to the vaccination status of the mice throughout the study. At 4 days post-infection, mice were euthanized by isoflurane overdose, and lungs were collected.

### Lung pathology

At the time of necropsy, lungs were evaluated for gross pathology using a 0-4-point gross lung discoloration scoring system [33]: 0 = normal, pink lungs; 1 = severe discoloration affecting less than 33% of the lung surface area or mild to moderate discoloration affecting less than 67% of the lung surface area; 2 = severe discoloration affecting 34% to 67% of the lung surface area or mild to moderate discoloration affecting 68% to 99% of the lung surface area; 3 = severe discoloration affecting 68% to 99% of the lung surface area or mild to moderate discoloration affecting 100% of the lung surface area; and 4 = severe discoloration affecting 100% of the lung surface area. Dark pink or gray lung color was considered mild or moderate discoloration, and red, maroon, or brown lung color was considered severe discoloration. The left lung lobe was inflated with and immersed in 10% neutral buffered formalin for 7 days, embedded in paraffin (Leica Paraplast), sectioned at 4μm thickness, and stained with hematoxylin and eosin (Richard Allan Scientific). Lung sections were blindly evaluated for pathological changes using two scoring systems previously validated for SARS-CoV-2-MA10 infection [31,32] in three 600X fields per tissue by an ACVP-boarded veterinary pathologist (S.A.M). Acute lung injury (ALI) scores were analyzed using the following parameters: A) neutrophils in the alveolar space: 0 = no cells, 1 = 1–5 cells, 2 = >5 cells; B) neutrophils in the interstitium: 0 = no cells, 1 = 1–5 cells, 2 = >5 cells; C) hyaline membranes: 0 = no membranes, 1 = 1 membrane, 2 = >1 membrane; D) proteinaceous debris in the airspaces: 0 = no debris, 1 = 1 instance, 2 = >1 instance; E) alveolar septal thickening: 0 = <2X thickness compared to mock-infected, 1 = 2-4X thickness compared to mock-infected, 2 = >4X thickness compared to mock-infected. ALI scores were calculated using the following formula: [(20 x A) + (14 x B) + (7 x C) + (7 x D) + (2 x E)] / 100 and averaged among the three fields. Diffuse alveolar damage (DAD) scores were determined using the following: 1 = no cellular sloughing or necrosis, 2 = occasional solitary cell sloughing and necrosis, 3 = >2 foci of cellular sloughing and necrosis with occasional septal wall hyalinization, and 4 = cellular sloughing and necrosis in >75% of the field and/or prominent hyaline membranes. The scores of the three fields were averaged to determine the final DAD score.

### Viral lung titers

Vero cells were plated at 2x10^5^ cells per well in 12-well plates and allowed to grow to 90–95% confluency overnight. Superior and middle lung lobes were homogenized in 0.5 mL of media (DMEM + 5% FBS + 1 mM L-glutamine) at 6000 rpm for 40 sec using a Roche MagNA Lyser homogenizer and centrifuged for 1 min at full speed to pellet debris. 50μL of the supernatant was added to 450 μL DMEM + 5% FBS + 1 mM L-glutamine media, and ten-fold serial dilutions were made to create a dilution series of 10^−1^ to 10^−6^. 200 μL of each homogenate dilution were added to the plated Vero cells and incubated at 37°C. After 1 h, 2 mL of overlay (50:50 mixture of 2.5% carboxymethylcellulose and 2X alpha MEM + 6% FBS + 2% penicillin/streptomycin + 2% L-glutamine + 2% HEPES) was added to each well, and plates were incubated at 37°C, 5% CO_2_ for 4 days. 2 mL of 4% paraformaldehyde was added to each well and allowed to fix cells overnight. Following removal of the fixative, wells were stained with 0.25% crystal violet, and visible plaques were counted and averaged between two technical replicate wells. Viral titers were calculated as pfu per lung tissue. The limit of detection (LOD) for the assay was determined to be 12.5 pfu / lung tissue, and samples that yielded no plaques were assigned a value of 6.25, half of the LOD.

### Statistical analyses

The values are presented as mean plus standard error of the mean (SEM). For Figs 1–3 and S3, a Student’s two-tailed *t*-test was performed to analyze the statistical differences among the groups. For Fig 5B, a two-way ANOVA with a Dunnett’s multiple comparisons post-test was performed. For Figs 5C and S5, a Kruskal-Wallis test with Dunn’s multiple comparisons was performed. *** *P* < 0.001; ** *P* < 0.01; * *P* < 0.05. The statistics were analyzed using GraphPad Prism 5 statistical software.

## Supporting information

S1 Fig Preparation of VLP nanoparticle, SARS-CoV-2 RBD, and VLP-based SARS-CoV-2 RBD vaccine. VLP nanoparticle (A), Fc-tagged SARS-CoV-2 RBD (B), and VLP-RBD complex (C) were each purified to high homogeneity. Left: representative elution profiles of the three proteins from Superose 6 Increase 10/300 GL high-resolution gel filtration chromatography. mAU: milli-absorbance unit at 280 nm wavelength. Right: representative SDS-PAGE gels (stained by coomasie blue) of peak fractions from the gel filtration chromatography. Experiments were repeated twice with similar results. (D) Stability of the VLP-RBD complex in the presence of competing antibodies in mouse serum. A protein pull-down assay was performed using biotinylated ACE2 (containing a His tag) as the bait. Only RBD-associated VLP (containing a His tag), but not free VLP or antibody-associated VLP, was pulled down from solution by the bait. The amount of pulled down VLP was correlated with the stability of the VLP-RBD complex.
(TIF)

S2 Fig Antibody responses induced by VLP-RBD vaccine after the prime immunization. Mouse sera from day 10 post-1^st^ immunization were examined for RBD-specific antibodies (A) and neutralizing antibodies against cell entry of pseudotyped SARS-CoV-2 (B). Mouse sera induced by VLP alone or the PBS buffer were also examined and compared to those induced by the vaccines. The experiments in (A) and (B) were performed in the same way as in Figs 2A and 4A, respectively, except that mouse sera from the prime immunization replaced those from the 2^nd^ immunization.
(TIF)

S3 Fig Antibody responses induced by VLP-RBD vaccine cross-neutralize the infections of SARS-CoV-1 and SARS-CoV-1-related bat coronavirus. The experiments were performed in the same way as in Fig 3A, except that SARS-CoV-1 and SARS-CoV-1-related bat coronavirus replaced SARS-CoV-2.
(TIF)

S4 Fig Representative images of flow cytometry showing that the mouse sera inhibit the interaction between SARS-CoV-2 RBD and human ACE2 receptor. The experiment was performed as described in Fig 3D. Median fluorescence intensity (MFI) values (blue lines) indicate inhibitory activity of sera (1:320 dilution) from mice immunized with RBD vaccine (A), VLP-RBD-M (B), VLP-RBD-E (C), or PBS (D). The higher the MFI values, the lower the inhibitory activity of the mouse sera. The interaction between SARS-CoV-2 RBD and ACE2 in the absence of mouse sera is shown in red line. The interaction between Fc fragment and ACE2 in the presence of mouse sera is shown in gray shades. Experiments were repeated twice with similar results.
(TIF)

S5 Fig More data on the protective efficacy of VLP-RBD vaccine in mice against SARS-CoV-2 challenge. Gross lung discoloration scores (A), ATS acute lung injury scores (B), and diffuse alveolar damage scores (C) of mice on day 4 are shown. The data are presented as mean ± SEM (n = 4–5 for mice in each group). A Kruskal-Wallis test with Dunn’s multiple comparisons was performed to analyze the statistical differences among the groups. **p < 0.01; *p < 0.05.
(TIF)

S1 Data All numerical values that were used to generate figures and supplementary figures. (XLSX)
