# Supplementary figures and images for: Novel virus-like nanoparticle vaccine effectively protects animal model from SARS-CoV-2 infection
Source: PLoS Pathog. 2021 Sep 7;17(9):e1009897. doi: 10.1371/journal.ppat.1009897 (PMC8448314; doi:10.1371/journal.ppat.1009897)

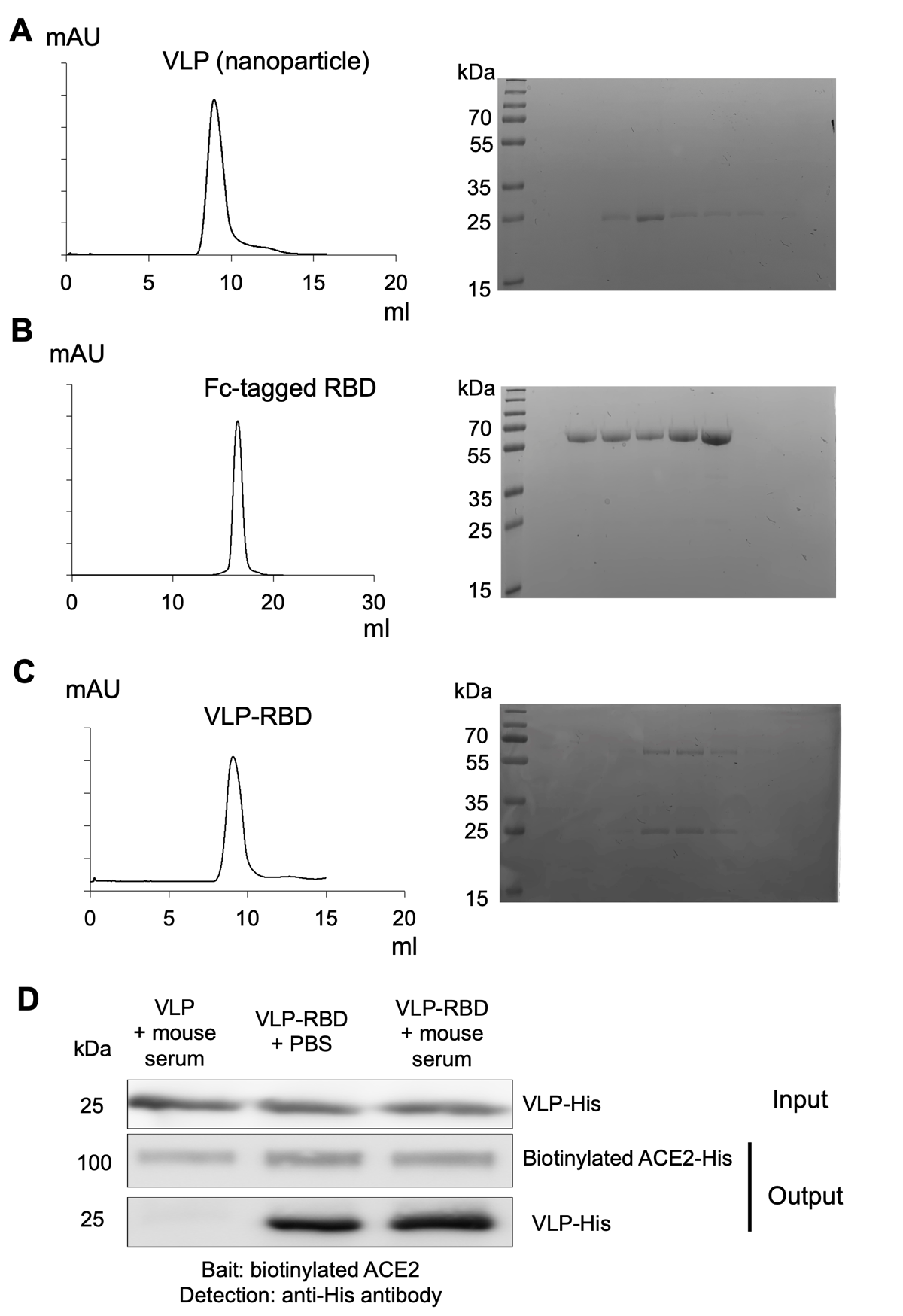

Supplement: S1 Fig — VLP nanoparticle (A), Fc-tagged SARS-CoV-2 RBD (B), and VLP-RBD complex (C) were each purified to high homogeneity. Left: representative elution profiles of the three proteins from Superose 6 Increase 10/300 GL high-resolution gel filtration chromatography. mAU: milli-absorbance unit at 280 nm wavelength. Right: representative SDS-PAGE gels (stained by coomasie blue) of peak fractions from the gel filtration chromatography. Experiments were repeated twice with similar results. (D) Stability of the VLP-RBD complex in the presence of competing antibodies in mouse serum. A protein pull-down assay was performed using biotinylated ACE2 (containing a His tag) as the bait. Only RBD-associated VLP (containing a His tag), but not free VLP or antibody-associated VLP, was pulled down from solution by the bait. The amount of pulled down VLP was correlated with the stability of the VLP-RBD complex. (TIF) [file ppat.1009897.s001.tif]

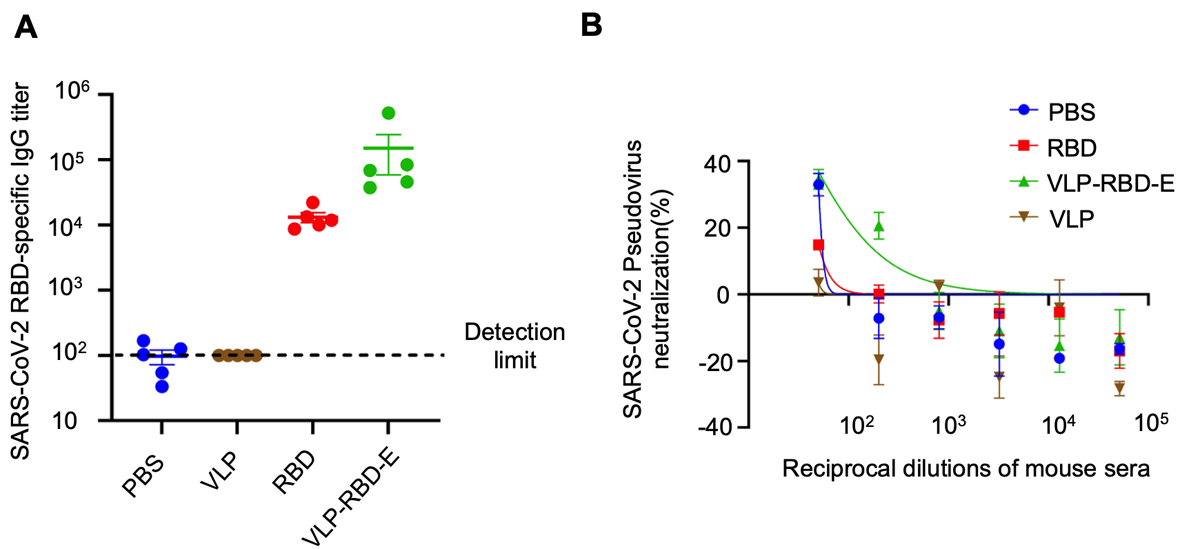

Supplement: S2 Fig — Mouse sera from day 10 post-1st immunization were examined for RBD-specific antibodies (A) and neutralizing antibodies against cell entry of pseudotyped SARS-CoV-2 (B). Mouse sera induced by VLP alone or the PBS buffer were also examined and compared to those induced by the vaccines. The experiments in (A) and (B) were performed in the same way as in Figs 2A and 4A, respectively, except that mouse sera from the prime immunization replaced those from the 2nd immunization. (TIF) [file ppat.1009897.s002.tif]

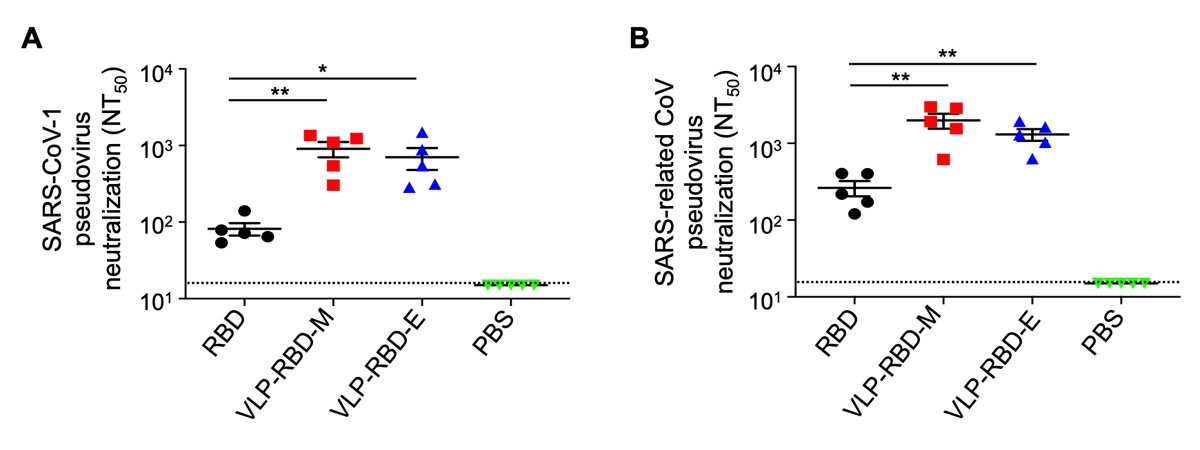

Supplement: S3 Fig — The experiments were performed in the same way as in Fig 3A, except that SARS-CoV-1 and SARS-CoV-1-related bat coronavirus replaced SARS-CoV-2. (TIF) [file ppat.1009897.s003.tif]

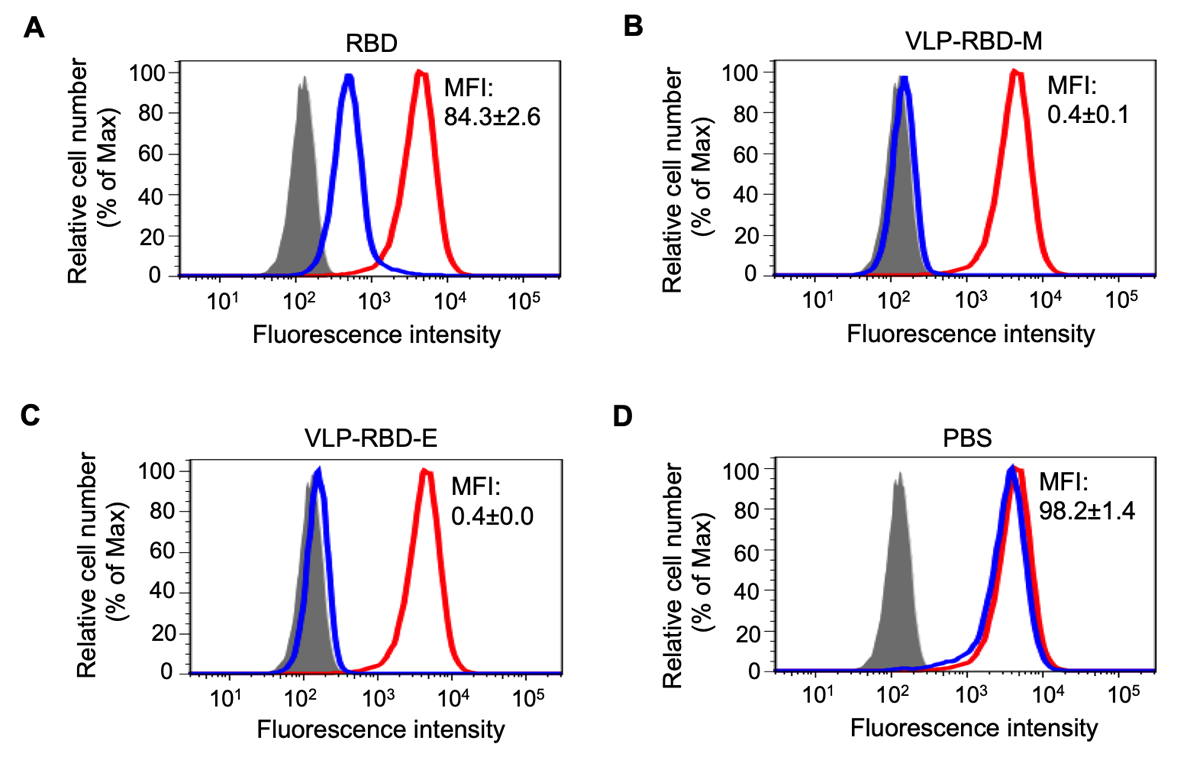

Supplement: S4 Fig — The experiment was performed as described in Fig 3D. Median fluorescence intensity (MFI) values (blue lines) indicate inhibitory activity of sera (1:320 dilution) from mice immunized with RBD vaccine (A), VLP-RBD-M (B), VLP-RBD-E (C), or PBS (D). The higher the MFI values, the lower the inhibitory activity of the mouse sera. The interaction between SARS-CoV-2 RBD and ACE2 in the absence of mouse sera is shown in red line. The interaction between Fc fragment and ACE2 in the presence of mouse sera is shown in gray shades. Experiments were repeated twice with similar results. (TIF) [file ppat.1009897.s004.tif]

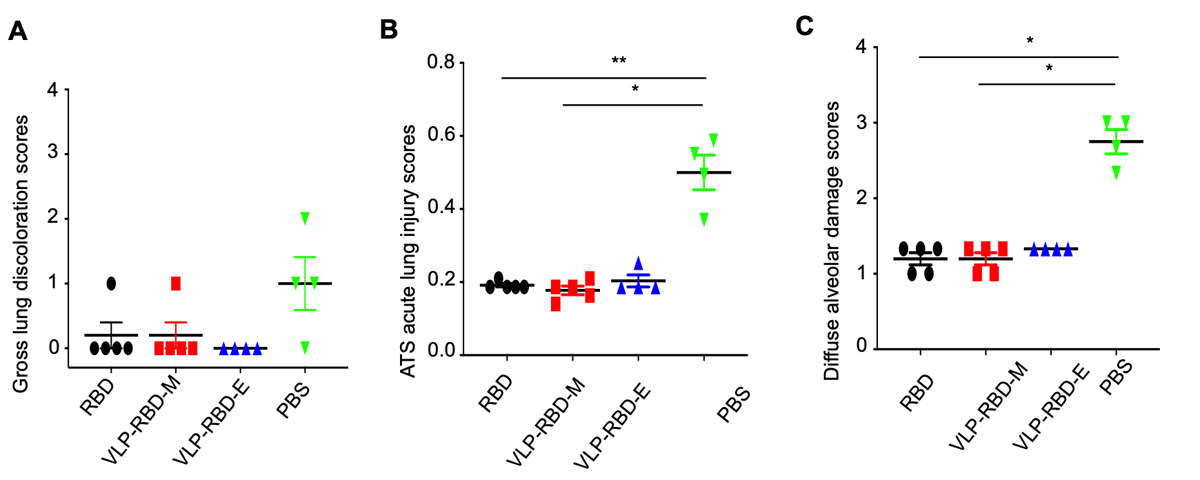

Supplement: S5 Fig — Gross lung discoloration scores (A), ATS acute lung injury scores (B), and diffuse alveolar damage scores (C) of mice on day 4 are shown. The data are presented as mean ± SEM (n = 4–5 for mice in each group). A Kruskal-Wallis test with Dunn’s multiple comparisons was performed to analyze the statistical differences among the groups. **p < 0.01; *p < 0.05. (TIF) [file ppat.1009897.s005.tif]
